# 2-(1,2,3,4-Tetra­hydro-1-naphth­yl)imidazolium chloride monohydrate

**DOI:** 10.1107/S1600536810030473

**Published:** 2010-08-18

**Authors:** Bruno Bruni, Gianluca Bartolucci, Samuele Ciattini, Silvia Coran

**Affiliations:** a Dipartimento di Scienze Farmaceutiche, Universitá di Firenze, Via U. Schiff 6, I-50019 Sesto Fiorentino, Firenze, Italy; b Centro di Cristallografia, Universitá di Firenze, Via della Lastruccia 3, I-50019 Sesto Fiorentino, Firenze, Italy

## Abstract

In the title compound, C_13_H_15_N_2_^+^·Cl^−^·H_2_O, the ions and water mol­ecules are ­connected by N—H⋯Cl, O—H⋯Cl, NH⋯Cl⋯HO, NH⋯Cl⋯HN and OH⋯Cl⋯HO inter­actions, forming discrete *D*(2) and *D*_2_^1^(3) chains, *C*_2_^1^(6) chains and *R*_4_^2^(8) rings, leading to a neutral two-dimensional network. The crystal structure is further stabilized by π–π stacking inter­actions [centroid–centroid distance = 3.652 (11) Å].

## Related literature

The title compound is a by-product obtained in the preparation of the popular decongestant tetra­hydro­zoline hydro­chloride and differs from the main product in the presence of an aromatic imidazole instead of a dihydro­imidazole group. For the structure of the tetra­hydro­zoline main product, see: Ghose & Dattagupta (1989 ▶); Ciattini *et al.* (2010 ▶). For the identification of the nature of the title compound, see: Bartolucci (2010 ▶). For hydrogen-bond motifs, see: Bernstein *et al.* (1995 ▶).
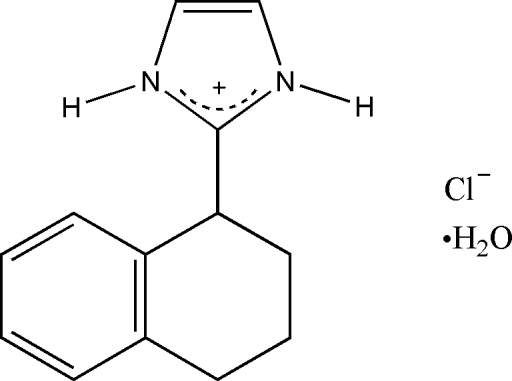


## Experimental

### 

#### Crystal data

C_13_H_15_N_2_^+^·Cl^−^·H_2_O
*M* *_r_* = 252.74
Monoclinic, 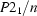

*a* = 9.8299 (1) Å
*b* = 12.6671 (2) Å
*c* = 10.5375 (2) Å
β = 92.666 (1)°
*V* = 1310.67 (3) Å^3^
*Z* = 4
Cu *K*α radiation
μ = 2.46 mm^−1^
*T* = 173 K
0.40 × 0.25 × 0.10 mm

#### Data collection

Oxford Diffraction Xcalibur PX Ultra CCD diffractometer
Absorption correction: multi-scan (*ABSPACK*; Oxford Diffraction, 2006 ▶) *T*_min_ = 0.594, *T*_max_ = 1.000
3831 measured reflections
1981 independent reflections
1883 reflections with *I* > 2σ(*I*)
*R*_int_ = 0.011
θ_max_ = 61.6°

#### Refinement

*R*[*F* ^2^ > 2σ(*F* ^2^)] = 0.033
*wR*(*F* ^2^) = 0.091
*S* = 1.09
1981 reflections
161 parameters
2 restraints
H atoms treated by a mixture of independent and constrained refinement
Δρ_max_ = 0.21 e Å^−3^
Δρ_min_ = −0.33 e Å^−3^

### 

Data collection: *CrysAlis PRO CCD* (Oxford Diffraction, 2006 ▶); cell refinement: *CrysAlis PRO CCD*; data reduction: *CrysAlis PRO RED* (Oxford Diffraction, 2006 ▶); program(s) used to solve structure: *SIR2004* (Altomare *et al.*, 1999 ▶); program(s) used to refine structure: *SHELXL97* (Sheldrick, 2008 ▶); molecular graphics: *ORTEP-3* (Farrugia, 1997 ▶); software used to prepare material for publication: *SHELXL97* and *PARST* (Nardelli, 1995 ▶).

## Supplementary Material

Crystal structure: contains datablocks global, I. DOI: 10.1107/S1600536810030473/bx2288sup1.cif

Structure factors: contains datablocks I. DOI: 10.1107/S1600536810030473/bx2288Isup2.hkl

Additional supplementary materials:  crystallographic information; 3D view; checkCIF report

## Figures and Tables

**Table 1:** Hydrogen-bond geometry (Å, °)

*D*—H⋯*A*	*D*—H	H⋯*A*	*D*⋯*A*	*D*—H⋯*A*
N1—H14⋯Cl^i^	0.88	2.21	3.0897 (16)	176
N2—H15⋯Cl^ii^	0.88	2.25	3.1164 (15)	169
O—H17⋯Cl	0.86 (1)	2.41 (1)	3.2612 (18)	173 (3)
O—H16⋯Cl^iii^	0.86 (1)	2.37 (1)	3.2252 (17)	175 (3)

Symmetry codes: (i) 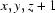; (ii) 
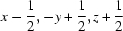
; (iii) 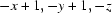.

**Fig. 1.**
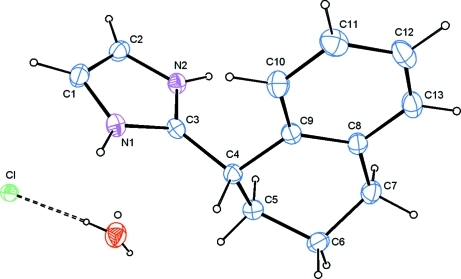
A view of the asymmetric unit of the title compound. Displacement ellipsoids are drawn at the 30% probability level. A hydrogen bond is denoted by a dashed line.The labelling criterion used for the structure of the related tetrahydrozoline hydrochloride has been preserved.

**Fig. 2.**
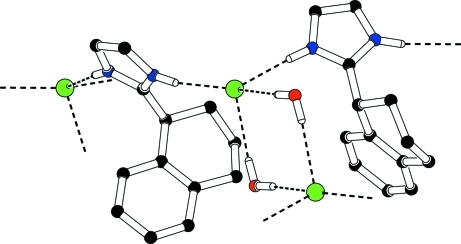
A view of the arrangement of hydrogen bonds in the structure of the title compound. Only hydrogen atoms involved in the formation of hydrogen bonds are shown for clarity. Hydrogen bonds are denoted by dashed lines.

## supplementary crystallographic information

### Comment

The industrial preparation of the widely used drug tetrahydrozolyne hydrochloride, 2-(1,2,3,4-tetrahydro-naphthalen-1-yl)-4,5- dihydro-1 *H*-imidazole hydrochloride, whose structure was reported several years ago (Ghose & Dattagupta, 1989), is generally accompanied by tiny amounts of an impurity (the title compound), whose nature has been identified (Bartolucci, 2010), but whose structure awaited investigation.

The trace byproduct, strictly related to the tetrahydrozolyne molecule, features an imidazole, rather than a dihydroimidazole ring. The structure of the present hydrochloride consists of cations, generated by *N*-protonation of the above imidazole derivative, chloride anions and solvate water molecules in 1:1:1 ratios. The content of the asymmetric unit is shown in Fig. 1. In the course of our investigations on these species, the structure of the main product of the preparation, already known (Ghose & Dattagupta, 1989), has been refined against low–temperature data and deposited at the Cambridge Structural database (deposition number CCDC 782942 [Ciattini *et al.*, (2010)]. In spite of the above difference between molecules of the main product and of the impurity and irrespective of different packing modes in the two structures, the overall molecular geometries are closely similar: *e.g.*, the dihedral angle formed by the best planes through the benzene and imidazole rings is 88.62 (5)° in the cation of the present structure and 88.00 (6)° in that of the tetrahydrozoline hydrochloride drug (170 K data).The structure of (I), C_13_H_15_N_2_^+^.Cl^–^ .H_2_O, comprises discrete ions and water molecules which are interconnected by N—H···Cl, O—H···Cl, NH···Cl···HO, NH···Cl···HN and OH···Cl···HO interactions to form discrete chains D(2), D_2_^1^(3); chains , C^1^_2_(6) and rings R_4_^2^(8) motifs (Bernstein *et al.*, 1995), leading to a neutral bidimensional- network. The crystal structure is further stabilized by π-π stacking interactions, (Cg1-Cg2= 3.652 (11) Å, α = 6.59 (10)° (Cg1 = N1/C1-C2/N2/C3 and Cg2 = C8-C13) and this interaction is not observed in the crystal structure of the tetrahydrozoline main product (Ghose & Dattagupta, 1989; Ciattini *et al.*, 2010).

### Experimental

Samples of the compound, made available from concomitant studies (Bartolucci, 2010), were obtained in suitable form for X-ray diffraction analysis by slow evaporation from methanolic solutions.

### Refinement

In the final refinement cycles non-hydrogen atoms were assigned anisotropic thermal parameters and H atoms had U_iso_(H) = 1.2 U_eq_(C, *N*), or U_iso_(H) = 1.5 U_eq_(O) for the water H atoms. Hydrogen atoms were placed in geometrically generated positions, riding, except for those of the water molecule, whose positions were refined with a restraint on O—H distances (0.86 (1) Å final value). Assigned C—H: tertiary CH 1.00 Å, secondary CH_2_ 0.99 Å, aromatic CH 0.95 Å. Aromatic N—H: 0.88 Å.

### Crystal data

**Table:**

C_13_H_15_N_2_^+^·Cl^−^·H_2_O	*F*(000) = 536
*M**_r_* = 252.74	*D*_x_ = 1.281 Mg m^−^^3^
Monoclinic, *P*2_1_/*n*	Cu *K*α radiation, λ = 1.54180 Å
Hall symbol: -P 2yn	Cell parameters from 3348 reflections
*a* = 9.8299 (1) Å	θ = 4.2–61.4°
*b* = 12.6671 (2) Å	µ = 2.46 mm^−^^1^
*c* = 10.5375 (2) Å	*T* = 173 K
β = 92.666 (1)°	Irregularly shaped prism, colourless
*V* = 1310.67 (3) Å^3^	0.40 × 0.25 × 0.10 mm
*Z* = 4	

### Data collection

**Table:**

Oxford Diffraction Xcalibur PX Ultra CCD diffractometer	1981 independent reflections
Radiation source: fine-focus sealed tube	1883 reflections with *I* > 2σ(*I*)
Oxford Diffraction, Enhance ULTRA assembly	*R*_int_ = 0.011
Detector resolution: 8.1241 pixels mm^-1^	θ_max_ = 61.6°, θ_min_ = 5.5°
ω scans	*h* = −11→6
Absorption correction: multi-scan (ABSPACK; Oxford Diffraction, 2006)	*k* = −14→12
*T*_min_ = 0.594, *T*_max_ = 1.000	*l* = −9→11
3831 measured reflections	

### Refinement

**Table:**

Refinement on *F*^2^	Secondary atom site location: difference Fourier map
Least-squares matrix: full	Hydrogen site location: inferred from neighbouring sites
*R*[*F*^2^ > 2σ(*F*^2^)] = 0.033	H atoms treated by a mixture of independent and constrained refinement
*wR*(*F*^2^) = 0.091	*w* = 1/[σ^2^(*F*_o_^2^) + (0.0459*P*)^2^ + 0.6835*P*] where *P* = (*F*_o_^2^ + 2*F*_c_^2^)/3
*S* = 1.09	(Δ/σ)_max_ < 0.001
1981 reflections	Δρ_max_ = 0.21 e Å^−^^3^
161 parameters	Δρ_min_ = −0.33 e Å^−^^3^
2 restraints	Extinction correction: *SHELXL97* (Sheldrick, 2008), Fc^*^=kFc[1+0.001xFc^2^λ^3^/sin(2θ)]^-1/4^
Primary atom site location: structure-invariant direct methods	Extinction coefficient: 0.0042 (10)

### Special details

**Table:**

Geometry. All s.u.'s (except the s.u. in the dihedral angle between two l.s. planes) are estimated using the full covariance matrix. The cell s.u.'s are taken into account individually in the estimation of s.u.'s in distances, angles and torsion angles; correlations between s.u.'s in cell parameters are only used when they are defined by crystal symmetry. An approximate (isotropic) treatment of cell s.u.'s is used for estimating s.u.'s involving l.s. planes.
Refinement. Refinement of *F*^2^ against ALL reflections. The weighted *R*-factor *wR* and goodness of fit *S* are based on *F*^2^, conventional *R*-factors *R* are based on *F*, with *F* set to zero for negative *F*^2^. The threshold expression of *F*^2^ > 2σ(*F*^2^) is used only for calculating *R*-factors(gt) *etc*. and is not relevant to the choice of reflections for refinement. *R*-factors based on *F*^2^ are statistically about twice as large as those based on *F*, and *R*- factors based on ALL data will be even larger.

### Fractional atomic coordinates and isotropic or equivalent isotropic displacement parameters (Å^2^)

**Table:**

	*x*	*y*	*z*	*U*_iso_*/*U*_eq_	
Cl	0.72704 (5)	0.40149 (4)	0.00302 (4)	0.0364 (2)	
N1	0.62280 (14)	0.24302 (12)	0.79789 (14)	0.0306 (4)	
H14	0.6564	0.2871	0.8558	0.037*	
N2	0.48201 (14)	0.15944 (11)	0.67424 (13)	0.0284 (4)	
H15	0.4056	0.1383	0.6354	0.034*	
C1	0.69789 (19)	0.17205 (15)	0.73092 (18)	0.0345 (4)	
H1	0.7937	0.1622	0.7383	0.041*	
C2	0.60936 (18)	0.11937 (15)	0.65322 (17)	0.0325 (4)	
H2	0.6305	0.0651	0.5951	0.039*	
C3	0.49175 (17)	0.23477 (13)	0.76179 (15)	0.0263 (4)	
C4	0.37597 (17)	0.29425 (14)	0.81497 (16)	0.0274 (4)	
H4	0.4159	0.3502	0.8723	0.033*	
C5	0.29045 (19)	0.34994 (15)	0.70929 (18)	0.0330 (4)	
H51	0.3418	0.4108	0.6771	0.040*	
H52	0.2720	0.3004	0.6378	0.040*	
C6	0.15650 (19)	0.38812 (15)	0.75959 (19)	0.0357 (5)	
H61	0.1051	0.4282	0.6925	0.043*	
H62	0.1749	0.4359	0.8328	0.043*	
C7	0.07197 (19)	0.29466 (16)	0.80081 (19)	0.0377 (5)	
H71	−0.0086	0.3211	0.8440	0.045*	
H72	0.0389	0.2549	0.7246	0.045*	
C8	0.15091 (18)	0.22119 (14)	0.88886 (17)	0.0300 (4)	
C9	0.29331 (18)	0.22005 (13)	0.89655 (16)	0.0275 (4)	
C10	0.3616 (2)	0.15248 (16)	0.98209 (17)	0.0359 (5)	
H10	0.4583	0.1524	0.9875	0.043*	
C11	0.2911 (2)	0.08543 (17)	1.05936 (19)	0.0424 (5)	
H11	0.3389	0.0398	1.1174	0.051*	
C12	0.1495 (2)	0.08569 (16)	1.05108 (19)	0.0417 (5)	
H12	0.0998	0.0399	1.1033	0.050*	
C13	0.0822 (2)	0.15226 (16)	0.96731 (18)	0.0380 (5)	
H13	−0.0146	0.1516	0.9623	0.046*	
O	0.46819 (17)	0.45111 (15)	0.17630 (17)	0.0595 (5)	
H16	0.415 (3)	0.487 (2)	0.125 (2)	0.089*	
H17	0.539 (2)	0.435 (3)	0.136 (3)	0.089*	

### Atomic displacement parameters (Å^2^)

**Table:**

	*U*^11^	*U*^22^	*U*^33^	*U*^12^	*U*^13^	*U*^23^
Cl	0.0348 (3)	0.0341 (3)	0.0390 (3)	−0.00069 (18)	−0.00995 (19)	0.00005 (18)
N1	0.0251 (8)	0.0341 (8)	0.0325 (8)	−0.0043 (6)	−0.0007 (6)	−0.0007 (7)
N2	0.0249 (8)	0.0308 (8)	0.0293 (8)	−0.0026 (6)	0.0009 (6)	−0.0017 (6)
C1	0.0259 (9)	0.0394 (10)	0.0384 (10)	0.0039 (8)	0.0045 (8)	0.0050 (9)
C2	0.0321 (10)	0.0330 (10)	0.0331 (10)	0.0061 (8)	0.0075 (8)	0.0021 (8)
C3	0.0262 (9)	0.0269 (9)	0.0259 (9)	−0.0048 (7)	0.0021 (7)	0.0025 (7)
C4	0.0253 (9)	0.0277 (9)	0.0294 (9)	−0.0031 (7)	0.0033 (7)	−0.0049 (7)
C5	0.0328 (10)	0.0299 (10)	0.0362 (10)	0.0012 (8)	0.0028 (8)	0.0013 (8)
C6	0.0330 (10)	0.0319 (10)	0.0419 (11)	0.0049 (8)	−0.0007 (8)	−0.0060 (8)
C7	0.0268 (9)	0.0419 (11)	0.0444 (11)	−0.0005 (8)	0.0021 (8)	−0.0083 (9)
C8	0.0289 (9)	0.0311 (9)	0.0306 (9)	−0.0048 (8)	0.0060 (7)	−0.0111 (8)
C9	0.0295 (9)	0.0283 (9)	0.0251 (9)	−0.0033 (7)	0.0046 (7)	−0.0065 (7)
C10	0.0324 (10)	0.0438 (11)	0.0318 (10)	−0.0018 (8)	0.0042 (8)	0.0012 (9)
C11	0.0528 (13)	0.0426 (12)	0.0323 (10)	−0.0025 (10)	0.0054 (9)	0.0043 (9)
C12	0.0505 (13)	0.0408 (11)	0.0351 (11)	−0.0170 (10)	0.0143 (9)	−0.0056 (9)
C13	0.0331 (10)	0.0427 (11)	0.0388 (11)	−0.0110 (9)	0.0098 (8)	−0.0126 (9)
O	0.0457 (10)	0.0654 (11)	0.0674 (11)	0.0046 (8)	0.0037 (8)	0.0186 (9)

### Geometric parameters (Å, °)

**Table:**

N1—C3	1.330 (2)	C6—H61	0.9900
N1—C1	1.378 (2)	C6—H62	0.9900
N1—H14	0.8800	C7—C8	1.504 (3)
N2—C3	1.328 (2)	C7—H71	0.9900
N2—C2	1.378 (2)	C7—H72	0.9900
N2—H15	0.8800	C8—C13	1.398 (3)
C1—C2	1.344 (3)	C8—C9	1.398 (3)
C1—H1	0.9500	C9—C10	1.392 (3)
C2—H2	0.9500	C10—C11	1.384 (3)
C3—C4	1.495 (2)	C10—H10	0.9500
C4—C9	1.532 (2)	C11—C12	1.390 (3)
C4—C5	1.535 (3)	C11—H11	0.9500
C4—H4	1.0000	C12—C13	1.369 (3)
C5—C6	1.521 (3)	C12—H12	0.9500
C5—H51	0.9900	C13—H13	0.9500
C5—H52	0.9900	O—H16	0.862 (10)
C6—C7	1.521 (3)	O—H17	0.861 (10)
			
C3—N1—C1	109.69 (15)	C7—C6—H61	109.6
C3—N1—H14	125.2	C5—C6—H62	109.6
C1—N1—H14	125.2	C7—C6—H62	109.6
C3—N2—C2	109.86 (15)	H61—C6—H62	108.1
C3—N2—H15	125.1	C8—C7—C6	112.62 (15)
C2—N2—H15	125.1	C8—C7—H71	109.1
C2—C1—N1	106.78 (16)	C6—C7—H71	109.1
C2—C1—H1	126.6	C8—C7—H72	109.1
N1—C1—H1	126.6	C6—C7—H72	109.1
C1—C2—N2	106.64 (16)	H71—C7—H72	107.8
C1—C2—H2	126.7	C13—C8—C9	117.99 (18)
N2—C2—H2	126.7	C13—C8—C7	120.10 (17)
N2—C3—N1	107.02 (15)	C9—C8—C7	121.90 (16)
N2—C3—C4	126.23 (15)	C10—C9—C8	119.65 (16)
N1—C3—C4	126.66 (15)	C10—C9—C4	119.22 (15)
C3—C4—C9	109.51 (14)	C8—C9—C4	121.11 (16)
C3—C4—C5	111.11 (14)	C11—C10—C9	121.25 (18)
C9—C4—C5	113.71 (14)	C11—C10—H10	119.4
C3—C4—H4	107.4	C9—C10—H10	119.4
C9—C4—H4	107.4	C10—C11—C12	119.2 (2)
C5—C4—H4	107.4	C10—C11—H11	120.4
C6—C5—C4	110.30 (15)	C12—C11—H11	120.4
C6—C5—H51	109.6	C13—C12—C11	119.65 (18)
C4—C5—H51	109.6	C13—C12—H12	120.2
C6—C5—H52	109.6	C11—C12—H12	120.2
C4—C5—H52	109.6	C12—C13—C8	122.23 (18)
H51—C5—H52	108.1	C12—C13—H13	118.9
C5—C6—C7	110.20 (15)	C8—C13—H13	118.9
C5—C6—H61	109.6	H16—O—H17	107 (3)
			
C3—N1—C1—C2	−0.4 (2)	C6—C7—C8—C9	−19.8 (2)
N1—C1—C2—N2	0.1 (2)	C13—C8—C9—C10	−0.9 (2)
C3—N2—C2—C1	0.2 (2)	C7—C8—C9—C10	178.28 (16)
C2—N2—C3—N1	−0.50 (19)	C13—C8—C9—C4	−179.19 (15)
C2—N2—C3—C4	−177.17 (16)	C7—C8—C9—C4	0.0 (2)
C1—N1—C3—N2	0.56 (19)	C3—C4—C9—C10	44.9 (2)
C1—N1—C3—C4	177.21 (16)	C5—C4—C9—C10	169.90 (16)
N2—C3—C4—C9	69.1 (2)	C3—C4—C9—C8	−136.78 (16)
N1—C3—C4—C9	−106.93 (19)	C5—C4—C9—C8	−11.8 (2)
N2—C3—C4—C5	−57.3 (2)	C8—C9—C10—C11	0.5 (3)
N1—C3—C4—C5	126.63 (18)	C4—C9—C10—C11	178.83 (17)
C3—C4—C5—C6	166.88 (15)	C9—C10—C11—C12	0.1 (3)
C9—C4—C5—C6	42.8 (2)	C10—C11—C12—C13	−0.2 (3)
C4—C5—C6—C7	−63.2 (2)	C11—C12—C13—C8	−0.2 (3)
C5—C6—C7—C8	51.0 (2)	C9—C8—C13—C12	0.8 (3)
C6—C7—C8—C13	159.38 (16)	C7—C8—C13—C12	−178.46 (17)

### Hydrogen-bond geometry (Å, °)

**Table:**

*D*—H···*A*	*D*—H	H···*A*	*D*···*A*	*D*—H···*A*
N1—H14···Cl^i^	0.88	2.21	3.0897 (16)	176.
N2—H15···Cl^ii^	0.88	2.25	3.1164 (15)	169.
O—H17···Cl	0.86 (1)	2.41 (1)	3.2612 (18)	173 (3)
O—H16···Cl^iii^	0.86 (1)	2.37 (1)	3.2252 (17)	175 (3)

Symmetry codes: (i) *x*, *y*, *z*+1; (ii) *x*−1/2, −*y*+1/2, *z*+1/2; (iii) −*x*+1, −*y*+1, −*z*.

## References

[bb1] Altomare, A., Burla, M. C., Camalli, M., Cascarano, G. L., Giacovazzo, C., Guagliardi, A., Moliterni, A. G. G., Polidori, G. & Spagna, R. (1999). *J. Appl. Cryst.* **32**, 115–119.

[bb2] Bartolucci, G. (2010). Private communication.

[bb3] Bernstein, J., Davis, R. E., Shimoni, L. & Chang, N.-L. (1995). *Angew. Chem. Int. Ed. Engl.* **34**, 1555–1573.

[bb4] Ciattini, S., Bruni, B., Bartolucci, G. & Coran, S. A. (2010). Private communication (number 1078) to the Cambridge Structural Database. Cambridge Crystallographic Data Centre, Cambridge, England.

[bb5] Farrugia, L. J. (1997). *J. Appl. Cryst.* **30**, 565.

[bb6] Ghose, S. & Dattagupta, J. K. (1989). *Acta Cryst. C**45***, 1522–1524.

[bb7] Nardelli, M. (1995). *J. Appl. Cryst.* **28**, 659.

[bb8] Oxford Diffraction (2006). *CrysAlis PRO CCD*, *CrysAlis PRO RED* and ABSPACK in *CrysAlis PRO RED* Oxford Diffraction Ltd, Abingdon, Oxfordshire, England.

[bb9] Sheldrick, G. M. (2008). *Acta Cryst.* A**64**, 112–122. 10.1107/S0108767307043930 18156677

